# Neural regeneration therapy after spinal cord injury induces unique brain functional reorganizations in rhesus monkeys

**DOI:** 10.1080/07853890.2022.2089728

**Published:** 2022-07-06

**Authors:** Jia-Sheng Rao, Can Zhao, Rui-Han Wei, Ting Feng, Shu-Sheng Bao, Wen Zhao, Zhaolong Tian, Zuxiang Liu, Zhao-Yang Yang, Xiao-Guang Li

**Affiliations:** a School of Biological Science and Medical Engineering, Beijing Key Laboratory for Biomaterials and Neural Regeneration, Beijing Advanced Innovation Center for Biomedical Engineering, Beihang University, Beijing, PR China; b Institute of Rehabilitation Engineering, China Rehabilitation Science Institute, Beijing, PR China; c Department of Neurobiology, School of Basic Medical Sciences, Capital Medical University, Beijing, PR China; d Department of Anesthesiology, Xuanwu Hospital Capital Medical University, Beijing, PR China; e State Key Laboratory of Brain and Cognitive Science, Institute of Biophysics, Chinese Academy of Sciences, Beijing, PR China; f Hefei Comprehensive National Science Center, Institute of Artificial Intelligence, Hefei, PR China; g Department of Biology, College of Life Sciences, University of Chinese Academy of Sciences, Beijing, PR China

**Keywords:** Nonhuman primate, neural regeneration, sensorimotor cortex, causal interactions, reorganization

## Abstract

**Purpose:**

Spinal cord injury (SCI) destroys the sensorimotor pathway and induces brain plasticity. However, the effect of treatment-induced spinal cord tissue regeneration on brain functional reorganization remains unclear. This study was designed to investigate the large-scale functional interactions in the brains of adult female Rhesus monkeys with injured and regenerated thoracic spinal cord.

**Materials and methods:**

Resting-state functional magnetic resonance imaging (fMRI) combined with Granger Causality analysis (GCA) and motor behaviour analysis were used to assess the causal interaction between sensorimotor cortices, and calculate the relationship between causal interaction and hindlimb stepping in nine Rhesus monkeys undergoing lesion-induced spontaneous recovery (injured, *n* = 4) and neurotrophin-3/chitosan transplantation-induced regeneration (NT3-chitosan, *n* = 5) after SCI.

**Results:**

The results showed that the injured and NT3-chitosan-treated animals had distinct spatiotemporal features of brain functional reorganization. The spontaneous recovery followed the model of “early intra-hemispheric reorganization dominant, late inter-hemispheric reorganization dominant”, whereas regenerative therapy animals showed the opposite trend. Although the variation degree of information flow intensity was consistent, the tendency and the relationship between local neuronal activity properties and coupling strength were different between the two groups. In addition, the injured and NT3-chitosan-treated animals had similar motor adjustments but various relationship modes between motor performance and information flow intensity.

**Conclusions:**

Our findings show that brain functional reorganization induced by regeneration therapy differed from spontaneous recovery after SCI. The influence of unique changes in brain plasticity on the therapeutic effects of future regeneration therapy strategies should be considered.

**Key messages**
- Neural regeneration elicited a unique spatiotemporal mode of brain functional reorganization in the spinal cord injured monkeys, and that regeneration does not simply reverse the process of brain plasticity induced by spinal cord injury (SCI).
- Independent “properties of local activity – intensity of information flow” relationships between the injured and treated animals indicating that spontaneous recovery and regenerative therapy exerted different effects on the reorganization of the motor network after SCI.
- A specific information flow from the left thalamus to the right insular can serve as an indicator to reflect a heterogeneous “information flow – motor performance” relationship between injured and treated animals at similar motor adjustments.

## 1. Introduction

Spinal cord injury (SCI), blocks the ascending and descending conduction pathways in the spinal cord, is a sudden and persistent interference in brain function, usually resulting in the functional “silence” of the corresponding cortex [1,2]. A large number of animal [3–5] and clinical [6] studies have demonstrated that the deafferented local cortical area is invaded by adjacent areas after SCI and are thus recruited to the sensation and movement of other intact parts. Recent resting-state functional magnetic resonance imaging (fMRI) studies on SCI further show that blocking afferent and efferent information induces alterations in the intensity and synchronization of spontaneous activity in the brain regions related to sensation, movement and inner consciousness [7,8]. The disintegration and remodelling of large-scale functional connections between cortexes [9–12] and between the cortex and subcortical nuclei [13,14] prove that SCI not only changes the specific activity of the regional cortex but also reshapes the connectivity pattern of brain functional networks.

Although many studies reported on the neuroplasticity changes in the brain elicited by the SCI- and spontaneous recovery-induced information flow, literature on the plasticity of brain function induced by intervention therapy after SCI is limited [15]. Some studies on peripheral nerve injury and treatment that provide evidence for assessing the effects of disruption and reconnection of afferent and efferent information flow on brain plasticity are available [16–18]. These studies demonstrated the reversal of cortical plastic reorganization after the recovery of afferent and efferent information flow, and the activation intensity and range of the “reawakening” local cortex region are improved by varying degrees [19].

Experimental researches on the treatment of SCI have developed recently. A variety of techniques, including exogenous stem/progenitor cell transplantation [20,21], Schwann cell transplantation [22], peripheral nerve transplantation [23] and biomaterial implantation [24–26], have been used to promote the regrowth of new nerve tissue in the lesion site where neurons/axonal bundles are missing. This effect is usually accompanied by a partial improvement in sensorimotor functions. Tissue regeneration, which reverses the loss of nervous tissue, is a physiological process contrary to that in SCI. In this case, the change in the process of brain functional reorganization is poorly understood.

Our previous work developed a neurotrophin-3(NT3)/chitosan carrier which released NT3 stably and constantly at least in 14 weeks, providing an optimal microenvironment for spinal cord regeneration [27] and eliciting robust de novo neural regeneration as well as sensorimotor functional recovery in both completely transected rats’ [25,28,29] and hemi-transected nonhuman primates’ thoracic cord [26]. Many myelinated, neurofilament (NF)-positive and biotinylated dextran amine (BDA)-fluorescein-positive fibres were observed in the rostral, middle, and caudal site of the NT3-chitosan tube after implantation [25]. With more than 12 months of NT3-chitosan material degradation cycle, corticospinal tract (CST) tracking with unilateral BDA injections in nonhuman primates showed a robust axonal regeneration across the damaged area to 15 mm caudal to the distal lesion edge [26]. These reconstructed neural tissues by NT3-chitosan material provide a substrate for the reconnection of afferent/efferent information flow and may influence brain functions. In this study, we used resting-state fMRI and Granger causality analysis (GCA) to evaluate the trends and differences of large-scale functional interactions in the brains of rhesus monkeys with injured and regenerated thoracic spinal cords. In addition, we further investigated the possible relationship between significantly changed causal interactions and the resting-state spontaneous activity of the local cortex and the hindlimb walking capability of animals. We hypothesized that given that the loss of nervous tissue could cause alterations in brain function, the regeneration should also lead to the reorganization of brain function and that the brain plasticity changes induced by these events had different spatiotemporal features, processes and relationship modes.

## 2. Materials and methods

### 2.1. Animal models

Nine female adult rhesus monkeys (*Macaca mulatta*, 4–6 years, 5 ± 1 kg) were used in this experiment. The animals were divided into the injured group (*n* = 4) and the NT3-chitosan group (*n* = 5) randomly. Numbers of animals were chosen to enable appropriate statistical testing and to ensure that a minimum of four animals were used per experimental group in each experiment. All animal experimental procedures were approved by the Biological and Medical Ethics Committee of Beihang University (approval no.: BM20180046), and the study conformed to the National Institute of Health – Office of Laboratory Animal Welfare (NIH-OLAW) guidelines for the care and use of laboratory animals.

Spinal cord T7-9 hemicord excision was performed in a dedicated animal operating room. Monkeys were anaesthetized by using Zoletil 50 (5 mg kg^−1^, intramuscular injection [im]) and xylazine hydrochloride (5 mg kg^−1^, im) and then maintained with sodium pentobarbital (20 mg kg^−1^ h^−1^, intravenous drop infusion [iv drop]). Before operation, the animals were intubated to ensure smooth breathing. Detailed surgical procedures were executed as previously described [26]. After laminectomy, the dura mater was cut under an operating microscope; a section of nervous tissue with a length of 10 mm on the right side of the thoracic cord was transected and removed; and the ventral side was scraped repeatedly with a blade to ensure that any residual fibres at the lesion site were removed. The animals in the NT3-chitosan group were transplanted with the NT3-chitosan scaffold into their injury areas, whereas those in the injured group did not receive any additional intervention. The dura, muscle and skin were subsequently sutured. Each animal was placed individually, and the environment was kept constant (temperature 24 ± 1 °C, humidity 40% ± 5%). After operation, antibiotic prophylaxis was provided once daily up to 3 d, and buprenorphine solution (50 µg, 100 *g*^−1^ body weight, once daily) was injected intramuscularly up to 5 d.

MRI examination was performed before injury (intact) and at 1, 2, 3, 6 and 12 months post-SCI. The kinematics-based gait test was not performed at 1 month after operation to prevent strenuous exercise from interfering with the recovery process [30]. The gait test was performed at other time points that were the same as those of MRI. All datasets were collected for further analyses.

### 2.2. NT3-chitosan tube fabrication

NT3-chitosan materials were fabricated *via* a previously described method [26,27,29]. Briefly, under sterile conditions, a 2% solution of poly-N-acetyl glucosamine derived from 85% deamidized chitosan (Sigma, St. Louis, MO) in 2% acetic acid was plasticised through treatment with 1 g of lithium chloride and 1 g of di (hydroxyethyl) sulphoxide with a melting point of 112 °C–113 °C. This mixture was stirred thoroughly. A capillary glass tube with a diameter of 2.0 mm was washed, autoclaved, dried, dipped vertically into the chitosan solution, slowly pulled out and placed vertically to volatilize the solvent. This process was repeated until the inner diameter of the tube reached 2 mm and the outer diameter of the tube reached 2–3 mm. The dried glass tube with chitosan was immersed in NaOH solution for 1 h and then in distilled water. The distilled water was replaced to keep the tube from becoming alkaline. A transparent chitosan tube was obtained by separating the glass capillary from the chitosan tube. The tube was cut to a length of 10 mm for experimental use, soaked in 75% alcohol and rinsed several times.

In an aseptic state, 10 mg of 85% deacetylated chitosan particles (Sigma, St. Louis, MO) was dissolved in 50 ml of deionized water, swelled for 6 h, and centrifuged. Then, the supernatant was discarded. The swollen particles were frozen at −20 °C for 24 h and maintained at 4 °C for 10 h. NT3 (Sigma, St. Louis, MO) was reconstituted to a concentration of 100 μg ml^−1^ in sterile cold deionized water. Then, 100 ng of NT3 was added to the swollen chitosan particle solution at 4 °C. The mixture was stirred at 4 °C for 6 h and then placed in a vacuum freeze dryer for freeze drying. The NT3-loaded dried chitosan particles were added to type I collagen solution at 4 °C and stirred for 30 min. The chitosan particles were collected *via* centrifugation. Then, 10 mg of the chitosan carrier containing 100 ng of NT3 was loaded into the chitosan tube. The tube was then stored at 4 °C.

### 2.3. MRI anaesthesia

Animals were anaesthetized with Zoletil 50 (5 mg kg^−1^, im) and xylazine hydrochloride (5 mg kg^−1^, im). Atropine sulphate injection (0.05 mg kg^−1^, im) was used to reduce bronchial and salivary secretion. Anaesthesia was maintained *via* the continuous administration of propofol (0.25 mg kg^−1^ min^−1^, iv drop) [31]. The level of anaesthesia was monitored periodically during scanning, with no somatic movement when pinching the toes, corneal reflex disappearance while the heart rate was kept above 70 times per min, and respiration rate exceeding 20 times per min as the standard [7,14].

### 2.4. MRI dataset acquisition

All MRI datasets were acquired on a Siemens 3 T MR scanner (MAGNETOM Skyra; Siemens). Brain data were collected by using a customized primate four-channel transmitter and receiver coil. Spinal cord data were obtained with a multichannel, fully dynamic parallel transmit array spine coil.

Brain functional data were obtained by using the gradient echo–echo planar imaging sequence with the following parameters: TR = 2000 ms, TE = 30 ms, field of view = 128 mm × 128 mm, matrix = 64 × 64, slice thickness 2 mm, flip angle = 90°. Twenty-five consecutive axial slices without gaps were used to cover the whole brain. Four seconds of empty scanning were added to avoid magnetic field heterogeneity at the beginning of the scanning. Each scanning period lasted for 4 min, and 120 volumes of EPI data were acquired. Structural data with the same centralization as functional data were obtained by using a three-dimensional magnetization prepared rapid acquisition gradient echo sequence with the following parameters: TR = 1520 ms, TE = 4.42 ms, flip angle = 15° and inversion time = 520 ms. The voxel spatial resolution was 1.0 mm × 0.5 mm × 0.5 mm and 180 contiguous slices were obtained to cover the entire brain.

Spinal cord structural images were obtained with a proton-density (PD) sequence. The imaging parameters were as follows: TR/TE = 3050 ms/11 ms, field of view = 192 mm × 192 mm, matrix = 320 × 320, slice thickness = 2 mm, flip angle = 147°, and 25 continuous axial slices covering five vertebral segments (T6–10). Same-centred diffusion tensor imaging (DTI) data were acquired through the single-shot spin-echo echo planar imaging sequence. A twice-refocusing pulse sequence was used to minimize eddy current effects. Axial-orientation diffusion-weighted images were acquired by using the following parameters: TR/TE = 4500 ms/104 ms, field of view = 196 mm × 196 mm, matrix = 128 × 128, slice thickness = 2 mm, *b* = 0 and 1000s/mm^2^, 13 gradient directions and 25 contiguous slices covering the lesion area. Data were acquired five times to enhance the signal-to-noise ratio. A Siemens generalized autocalibrating partially parallel acquisition imaging system was used with an acceleration factor of 4 to reduce the extent of susceptibility artefacts. Readout bandwidth was set to 1396 Hz, and the echo train spacing was set to 0.82 ms. The saturated band was set in the chest and abdominal cavity to decrease physiological motion artefacts [32].

### 2.5. Functional data processing

Brain functional data were processed by using SPM8 (http://www.fil.ion.ucl.ac.uk/spm). The detailed process method is described in previous studies [7,8,14]. Briefly, the preprocessing stage included slice time delay compensation, head movement correction (maximum head displacement <1 mm, maximum rotation <1°), spatial normalization with INIA19 Primate Brain Atlas [33] and smoothing (FWHM = 3 mm). The first 10 volumes of every scan were excluded to avoid the possible instability of the initial MRI signal. A temporal band-pass filter (0.0025–0.05 Hz) was used to remove low-frequency drifts and high-frequency physiological noise [34]. On the basis of the INIA19 Primate Brain Atlas and research by [35], the cerebellum (Cb), lateral primary somatosensory cortex (S1l), medial primary somatosensory cortex (S1m), secondary somatosensory cortex (S2), parieto-occipital association cortex (PE area), lateral primary motor cortex (M1l), medial primary motor cortex (M1m), supplementary motor area (SMA), middle frontal gyrus (MFG), insular (Ins), putamen (Pu), thalamus (Th), and middle occipital gyrus (MOG) were manually obtained from the bilateral monkey brain with 26 regions of interest (ROIs) for the calculation of GCA, fractional amplitude of low-frequency fluctuation (fALFF) and regional homogeneity (ReHo).

### 2.6. GCA calculation

The averaged time series of all voxels in each ROI were extracted. The causal interactions between the 26 ROIs of each animal at each time point were calculated by using multivariate conditional GCA in Resting-state fMRI Data Analysis Toolkit (REST V1.8, http://www.restfmir.net). Then, the GCA matrix of sensorimotor-related networks was formed for subsequent inter- and intra-group analyses. Longitudinal changes were calculated in each group. The number of subcortical brain areas and the ratio of GCA originating from the left brain regions to GCA in the whole brain involved in each group at each timepoint were obtained. The number of intra- and inter-hemispheric altered GCA in both groups was acquired separately in the early (1–3 months post-SCI) and late (6–12 months post-SCI) stages and normalized by that in the late stage as a standard.

### 2.7. FALFF and ReHo calculations

The fALFF and ReHo of blood oxygenation level-dependent (BOLD) signals in each ROI were calculated for subsequent correlation analyses. The fALFF and ReHo values of each animal were divided by its own mean value of the whole brain for standardization to decrease the effect of individual variability [7,8]. Data calculation was carried out in Data Processing Assistant for Resting-state fMRI (DPARSFA V2.3, http://www.restfmir.net).

### 2.8. DTI data processing

DTI datasets were processed and analysed by using dedicated MedINRIA software (http://www-sop.inria.fr/asclepios/software/MedINRIA). The detailed methods have been reported in previous articles [32,36]. In brief, image post-processing mainly included eddy current distortion correction and geometric distortion correction. For eddy current distortion, five b0 images in EPI dataset were averaged as a reference. All diffusion-weighted images in EPI datasets were coregistered to the reference by using linear affine transformation. For geometric distortion, the averaged b0 image was registered to PD volume through the diffeomorphic demons’ registration algorithm to obtain a nonrigid displacement field. The deformation vector field was extracted and then applied to all EPI data to complete the geometric distortion correction.

### 2.9. Fibre tracking

Eigenvalues in three perpendicular directions were extracted from each pixel of each animal to calculate the fractional anisotropy [37]. The directions of the eigenvectors that were related to the largest eigenvalues were set to the main direction of local neural fibres [38]. The threshold of fibre-tracking background removal was set to 0.10 to exclude nonpositive voxels and noise. Interpolated fibre smoothing was set to 20%, and the minimum fibre length was set to 10 mm to obtain continuous fibre reconstruction [26]. To quantify the possible regenerated fibres, the number of fibres at the caudal end of the injury was gained in injured and NT3-chitosan treated animals at each timepoint. To decrease the effect of individual variability, the number of fibres obtained from each animal was divided by its own number of fibres at the remote normal-like spinal cord to standardize (i.e. the ratio of regenerated fibres to remote normal-like fibres was calculated).

### 2.10. Kinematic analysis of locomotion

The hindlimb gait datasets of each animal were acquired by using a Vicon system (Vicon 8, Oxford Metrics Limited Company, UK). Gait test, data collection, processing and calculation were performed as previously described [39–42]. In brief, reflective markers were fixed on the anterior and posterior superior iliac spines, 2/3 of the femur, knee joint, tibia midpoint, ankle joint, heel and the second metatarsophalangeal joint in the right hindlimb. Eight infra-red cameras were used to record the three-dimensional position of the reflective markers during stepping. Animals were walking bipedally on a treadmill at a speed of 0.22 m *s*^−1^ (recording frequency: 100 Hz) while their upper body was restrained. At least 10 steps of each animal at each timepoint were obtained for subsequent processing and analysis. Gait cycle duration, stance duration, stride length, step height, swing endpoint velocity, hip joint flexion, hip joint extension, hip joint amplitude, knee joint flexion, knee joint extension, knee joint amplitude, ankle joint flexion, ankle joint extension and ankle joint amplitude were calculated by using Matlab (MathWorks, Natick, MA). Principal component analysis (PCA) was adopted to process the above gait parameters and reconstruct PCs 1–5 in a single gait cycle. The extent of the change in motor function is the sum of the square deviation (SSD) between the PC1–5 values of each animal at each timepoint post-SCI and the PC1–5 values of intact status.

### 2.11. Statistical analyses

Statistical analyses were conducted using SPSS 20.0 (SPSS, Chicago, IL, USA). All data were shown as mean ± SEM. The Shapiro–Wilk method was used for data normality analysis, and the Levene test was applied to determine the homogeneity of variance. An independent t-test was used to detect the difference of the ratio of regenerated fibres to remote normal-like fibres between the two groups at all timepoints (six-time points, 6 tests, the significance level was set at *p* ≤ 0.05 with Bonferroni multiple comparisons, uncorrected *p* ≤ 0.05/6 = 0.0083). The paired t-test was carried out to detect the difference of GCA matrices between intact and post-SCI (intact *vs.* 1 month, intact *vs.* 2 months, intact *vs.* 3 months, intact *vs*. 6 months and intact *vs.* 12 months, all after operation) in injured and NT3-chitosan groups, individually. The significance level was set at *p* ≤ 0.05 with Bonferroni multiple comparisons (uncorrected *p* ≤ 0.05/5 = 0.01). The independent t-test and the Chi-square test were executed separately to detect the difference in the proportion of subcortical regions involved in the reorganization, the proportion of GCA originating from the left brain regions, and the proportion of intra- and inter-hemispherical GCA numbers between the injured and NT3-chitosan groups. The significance level was set at *p* ≤ 0.05.

To investigate the effect of time (five time points) × group (two groups) interaction on the GCA matrices, two-way ANOVA followed by Bonferroni test was used. Significantly altered GCA information flows under time × group interactions were obtained, an independent t-test was used to detect the difference of flow intensity changes between the two groups at the post-SCI stage (five time points, five tests, the significance level was set at *p* ≤ 0.05 with Bonferroni multiple comparisons, uncorrected *p* ≤ 0.05/5 = 0.01); paired t-test was used to examine the difference of flow intensity changes among 1, 2, 3, 6 and 12 months post-SCI in both groups, individually (five time points, 10 tests in each group, uncorrected *p* ≤ 0.05/10 = 0.005).

Correlations between significantly changed GCA intensities and the fALFF/ReHo values in the local brain region were calculated by using Pearson correlation analysis. The significance level was set at *p* ≤ 0.05. The Chow-test was performed to analysis the discrepancy between the injured and NT3-chitosan groups in the correlation modes. The significance level was set at *p* ≤ 0.05 with Bonferroni multiple comparisons (two tests, uncorrected *p* ≤ 0.05/2 = 0.025).

To explore the difference of gait PC and SSD scores between the injured and NT3-chitosan groups, an independent t-test was executed at all times after SCI. The level of significance was set at *p* ≤ 0.05, and was adjusted for multiple testing using the Bonferroni correction (four time points, four tests, uncorrected *p* ≤ 0.05/4 = 0.0125). Relationships between the significantly changed GCA intensities and the gait PC1–5 scores were calculated by using Spearmen correlation analysis. The significance level was set at *p* ≤ 0.05. Chow-test was used to detect the variation between the injured and NT3-chitosan groups in the correlation modes. The significance level was set at *p* ≤ 0.05. To identify the relationship between the fibre tracking results and gait performances, Pearson (normality) or Spearmen (non-normality) correlation analysis was implemented between the ratio of regenerated fibres to remote normal-like spinal cord fibres and SSD scores. The significance level was set at *p* ≤ 0.05.

## 3. Results

**Figure 1.**
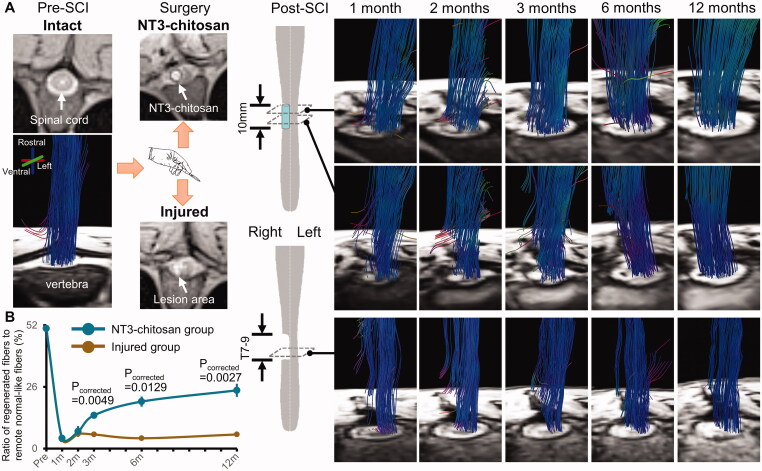
Representative DTI-fibre tracking longitudinal results of injured and NT3-chitosan treated animals. Data processing just following the same methods as we previously reported [26]. (A) Reconstructed fibre bundles were superimposed on the corresponding axial PD weighted structural images. Before operation, the structure of the spinal cord exhibited good integrity, and the fibre bundles filled the whole spinal cord structure in an orderly manner. After operation, the tissue of the right thoracic cord was damaged and the structural integrity was destroyed, and the implanted NT3–chitosan scaffold could be clearly observed. The fibres (rostrocaudal orientation) gradually extended across the surgical site over time, reconnecting the rostral and caudal ends of the injured cord in the regenerative therapy animals. On the axial structural images, the material boundary gradually blurred and disappeared with the degradation of the implanted NT3-chitosan scaffold. In injured animals, no fibre bundles were present within the lesion and passed through the injured site. (B) The proportion of the regenerated fibres to the number of remote normal-like spinal cord fibres was significantly different between the two groups at 3, 6, and 12 months post-SCI (Independent t-test with Bonferroni multiple comparisons). m, months.

In our previous studies, robust axonal regeneration after thoracic SCI was achieved by implanting NT3-chitosan bioactive material scaffolds in rodents [29] and primates [26]. Consistent with our previously reports, NT3-chitosan-treated rhesus monkeys in this study also showed spinal neural fibre regeneration as expected. The longitudinal observation of MRI-DTI displayed that the MRI signal of the regrowth tissue appeared in the lesion area and connected the rostral and caudal regions over time. By contrast, the injured animals demonstrated gradual spinal cord atrophy and no sign of regeneration (Figure 1(A)). The ratio of regenerated fibres obtained from the treated animals was obviously higher than that from the injured group at 3, 6 and 12 months post-SCI (*p*_corrected_ <0.0130) (Figure 1(B)).

**Figure 2.**
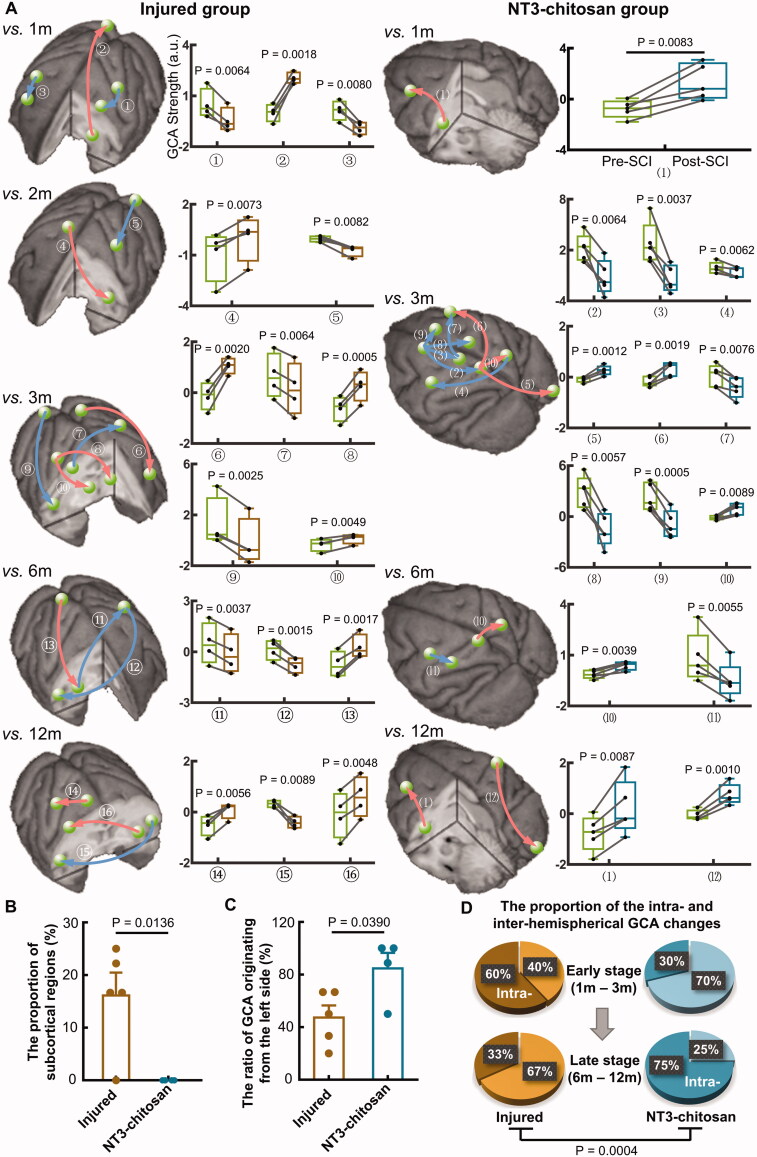
The injured and NT3-chitosan-treated animals have different brain functional reorganization features. (A) Causal interactions that significantly deviated from intact status in each timepoint post-SCI were superimposed on the three-dimensional monkey brain. The corresponding quantitative assessment described the alteration of intensity (Paired t-test with Bonferroni multiple comparisons). Red indicates the GCA intensity that is significantly higher than the intact state, whereas blue indicates the opposite. Box plots show the median and 25th and 75th percentiles; whiskers indicate the minimum and maximum values. Raw data were shown in graphs. The green ball represents both ends of the brain regions of the causal interaction, and the arrows indicate the direction of information flow. (B) Significant difference between the two groups in the proportion of subcortical regions involved in the reorganization (Independent t-test). (C) Notable difference between the two groups in the ratio of GCA originating from the left brain regions (Independent t-test). Data dots represent the proportion at each timepoint post-SCI. (D) The two group animals differed in the proportions of intra- and inter-hemispheric GCA alterations at early and late stages (Chi-square test). L: left; R: right; m: months; S1l: lateral primary somatosensory cortex; M1l: lateral primary motor cortex; Pu: putamen; MOG: middle occipital gyrus; MFG: middle frontal gyrus; M1m: medial primary motor cortex; Ins: insula; PE area: parieto-occipital association cortex; Th: thalamus; S2: secondary somatosensory cortex; SMA: supplementary motor area; S1m: medial primary somatosensory cortex. Injured group: ①L.S1l→L.M1l, ②L.Pu→R.MOG, ③R.M1l→R.MFG, ④R.M1m→L.Ins, ⑤L.MOG→L.S1l, ⑥L.PE area→L.MFG, ⑦R.M1l→L.S1l, ⑧R.S1l→L.Th, ⑨R.MOG→R.S2, ⑩R.S1l→R.Th, ⑪R.Pu→L.S1l, ⑫L.S1l→R.S2, ⑬R.PE area→R.Pu, ⑭R.M1m→R.S1l, ⑮L.S2→R.S2, ⑯L.Pu→R.M1l. NT3-chitosan group: (1)L.Ins→L.MFG, (2)L.SMA→L.S1m, (3)L.SMA→L.M1m, (4)R.PE area→L.M1l, (5)L.S1m→R.MOG, (6)L.S1m→R.MFG, (7)L.M1m→R.MFG, (8)L.SMA→R.M1m, (9)L.SMA→R.SMA, (10)L.S1m→R.PE area, (11)L.M1l→L.S1l, (12)R.S1l→R.MOG.

We used GCA to detect cortical–cortical and cortical–subcortical information flows to explore the effect of nervous tissue defects and regeneration on functional interactions among brain regions. GCA not only provides the intensity of information flow between two cortical regions but also shows the direction, which is of great importance for revealing the origin of changed information flows in brain functional networks [43]. At each time point post-SCI, GCA flows which significantly deviated from intact status were obtained from GCA matrices (Figure 2(A)). The changed interactions in the NT3-chitosan group had unique spatiotemporal characteristics. Compared with those in the treated animals, abnormal causal interactions in the injured primates involved a large number of subcortical regions (*p* = 0.0136) (Figure 2(B)). NT3-chitosan-treated animals had a higher ratio of GCA flows originating from the left hemispherical regions than that in injured ones (*p* = 0.0390) (Figure 2(C)). In the injured group, intra-hemispherical GCA changes were the main form of brain functional reorganization in the early stage after operation, whereas inter-hemispherical GCA alterations dominated in the late stage. In contrast, most of the inter-hemispherical interactions in the NT3-chitosan-treated animals altered significantly in the early stage, whereas most changes in the late stage were mainly in the intra-hemispheres (*vs.* injured group, *p* = 0.0004) (Figure 2(D)). In addition, most GCA alterations in the treated animals were concentrated at 3 months post-SCI (75%), and the left side supplementary motor area (L.SMA), as the origin of the interactions, exhibited the highest number of modifications (Figure S1).

**Figure 3.**
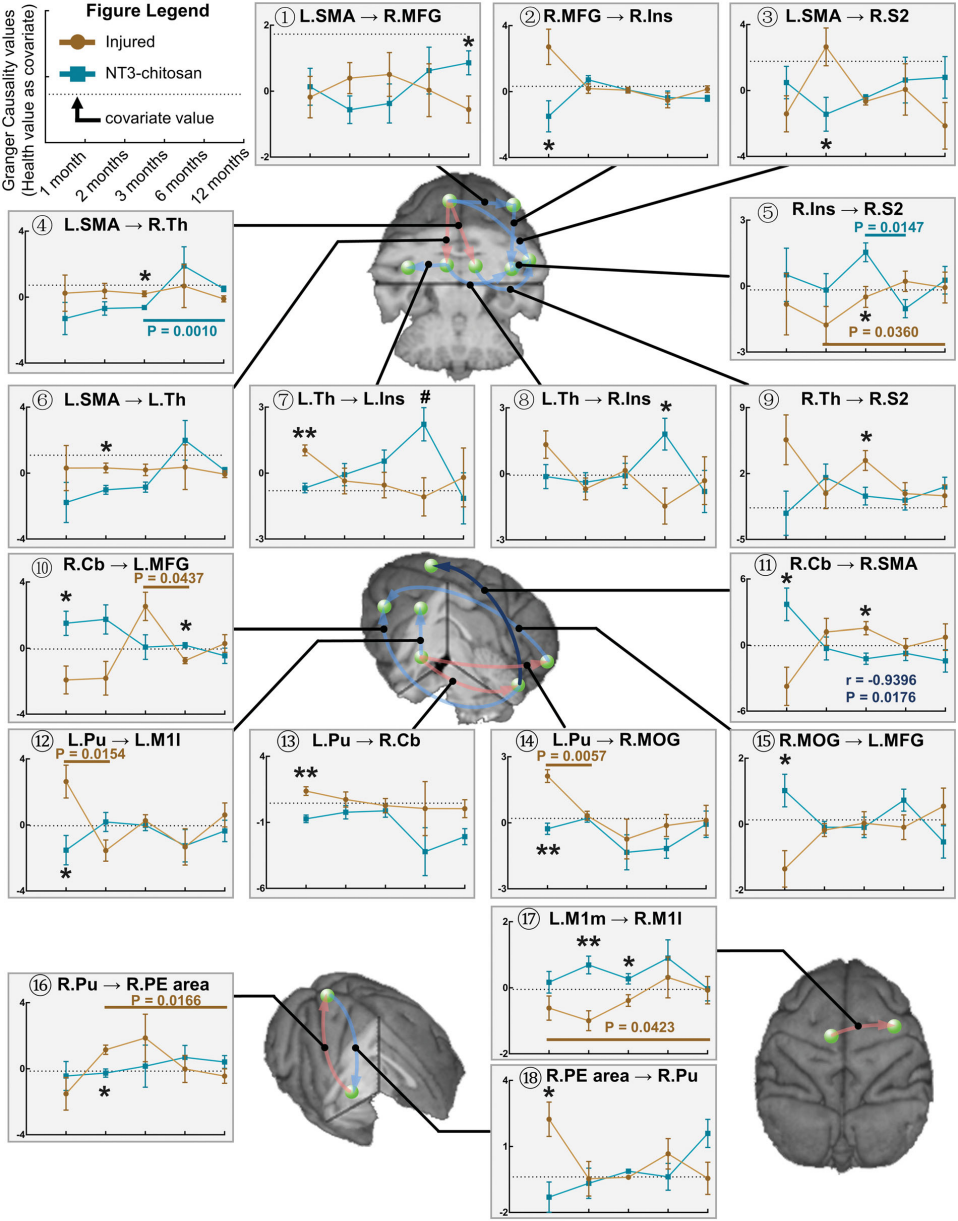
Brain reorganization processes are mostly independent between the injured and NT3-chitosan-treated groups. Granger Causality connections that were significantly affected by time × group interaction were overlaid on the three-dimensional monkey brain (repeated-measures ANOVA, for detailed values see Supplementary Table 1). Quantitative evaluation was performed to describe the longitudinal variation in the corresponding GCA intensity with time. Light red/blue indicates positive/negative correlations (but not significant) in GCA intensity between the two groups, and dark blue indicates a prominent negative relationship (information flow R.Cb→R.SMA, *r* and *p* values are given). Single-factor principal effect analysis showed that the intra-group GCA intensity of injured (brown) and NT3-chitosan (dark cyan) animals altered remarkably among different time points (*p* values have been given). Inter-group analysis displayed significant differences in GCA intensity at a specific timepoint. Data are presented as mean ± SEM. *p < 0.05; **p < 0.01 (for detailed *p* values see Supplementary Table 1). L: left; R: right; SMA: supplementary motor area; MFG: middle frontal gyrus; Ins: insula; S2: secondary somatosensory cortex; Th: thalamus; Cb; cerebellum; Pu: putamen; M1l: lateral primary motor cortex; MOG: middle occipital gyrus; PE area: parieto-occipital association cortex; M1m: medial primary motor cortex.

Then, we further explored time × group interaction effects for all GCA connections when the intact value was taken as covariable. Significantly altered GCA flows were obtained by using two-way ANOVA analysis (Figure 3, Supplementary Table 1). Although multiple information flows from the L.SMA, left side putamen (L.Pu), and left side medial primary motor cortex (L.M1m) were affected, none of the information flows sent to these three brain regions were modulated by time × group interactions. These three brain regions are involved in the action planning, regulation, integration, management, and execution of the paralysed right hindlimb, showing the susceptibility of exercise-related brain regions to the change of physiological states in the spinal cord and the critical role in initiating the reorganization of functional networks. The correlation analysis of the changes in the above GCA values showed that the change trends of most information flows were not correlated between the two groups, and only the information flow from the right side cerebellum (R.Cb) to R.SMA showed a significantly negative correlation (Figure 3), which revealed the independent brain function reorganization processes between the injured and NT3-chitosan animals.

**Figure 4.**
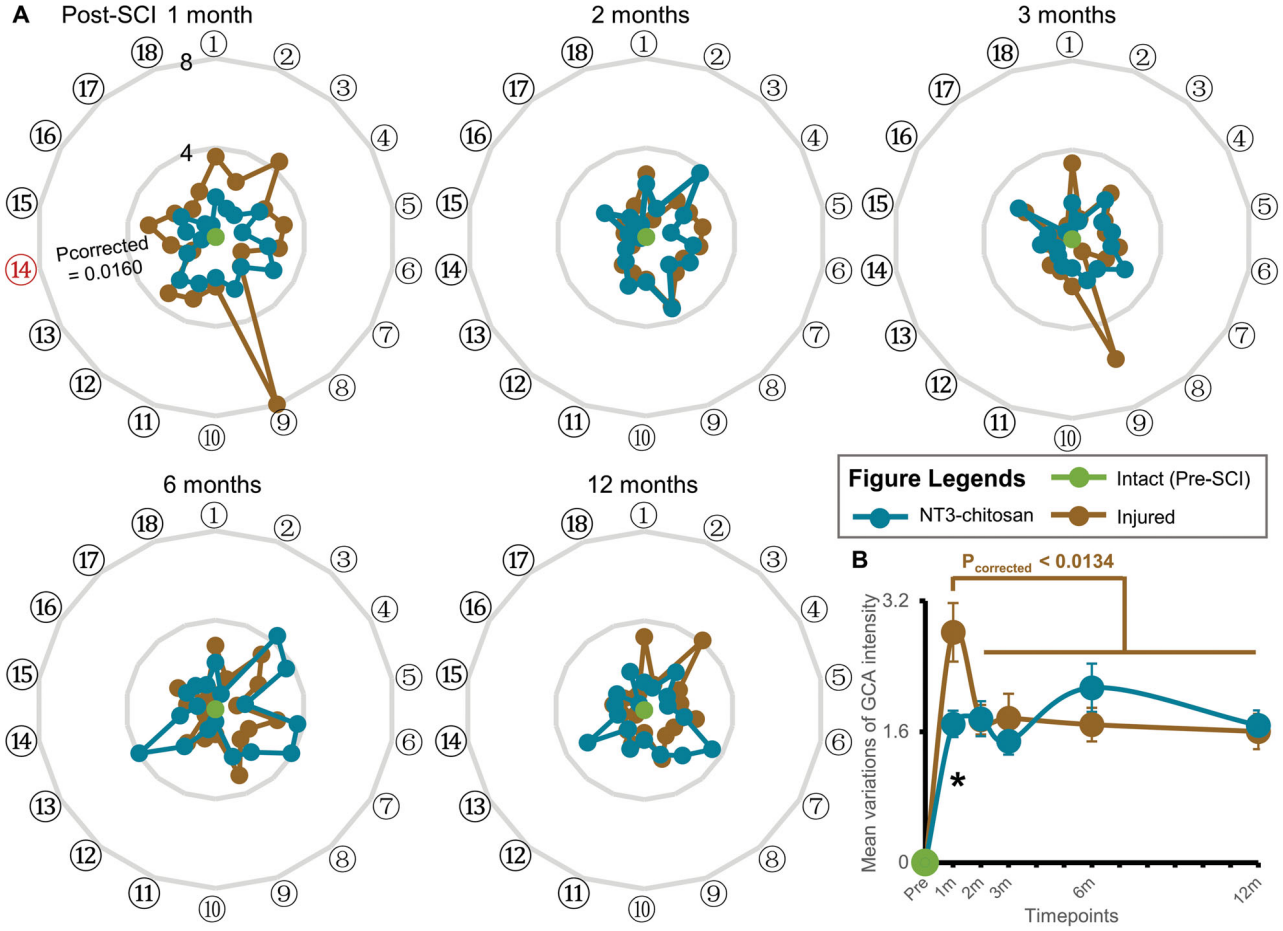
Intensity changes of brain causal interactions between the injured and NT3-chitosan groups are basically the same. (A) Radar map showing the extent of GCA changes which were significantly affected by time × group interaction. Order numbers were corresponded to the ones also shown in Figure 3. The red serial number indicates a noteworthy difference in variation degree between the two groups (independent t-test with Bonferroni multiple comparisons). (B) Change extent and tendency of GCA intensity with time in the two groups (intra-group: paired t-test with Bonferroni multiple comparisons; inter-group: independent t-test with Bonferroni multiple comparisons, *p_corrected_ = 0.0369). L: left; R: right; SMA: supplementary motor area; MFG: middle frontal gyrus; Ins: insula; S2: secondary somatosensory cortex; Th: thalamus; Cb: cerebellum; Pu: putamen; M1l: lateral primary motor cortex; MOG: middle occipital gyrus; PE area: parieto-occipital association cortex; M1m: medial primary motor cortex. Order numbers: ①L.SMA→R.MFG, ②R.MFG→R.Ins, ③L.SMA→R.S2, ④L.SMA→R.Th, ⑤R.Ins→R.S2, ⑥L.SMA→L.Th, ⑦L.Th→L.Ins, ⑧L.Th→R.Ins, ⑨R.Th→R.S2, ⑩R.Cb→L.MFG, ⑪R.Cb→R.SMA, ⑫L.Pu→L.M1l, ⑬L.Pu→R.Cb, ⑭L.Pu→R.MOG, ⑮R.MOG→L.MFG, ⑯R.Pu→R.PE area, ⑰L.M1m→R.M1l, ⑱R.PE area→R.Pu.

We calculated the extent to which the intensity of above GCA deviated from the normal value in each animal at each timepoint. The degree of change in each information flow during the reorganization processes was compared between the two groups (Figure 4(A)). No significant difference between the injured and NT3-chitosan groups was observed at most timepoints, indicating that although brain functional reorganization differed, the overall intensity alterations of the brain causal interaction were the same. The averaged degree of change in information flows’ intensity in the injured group showed an unusually high peak at 1-month post-SCI (*p*_corrected_ <0.0134) and then decreased significantly and remained stable from 2 months after operation (Figure 4(B)). Since recovery and plasticity are maximized in the acute phase after SCI, this result is in line with our expectations. However, the spike impact on the stability of the brain functional network was not observed in the NT3-chitosan animals (*vs.* injured group, *p*_corrected_ =0.0369). Altered GCA intensity in the NT3-chitosan group remained stable after operation (*p*_corrected_ >0.05). This phenomenon may be mainly due to NT3–chitosan implantation in the injured area, but further pharmacological studies are needed. In addition, the degree of changes in brain interactive connections in both groups did not return to normal at the end of the experiments, suggesting that the brain needs strengthened causal interactions among regions with complete related functional activities.

**Figure 5.**
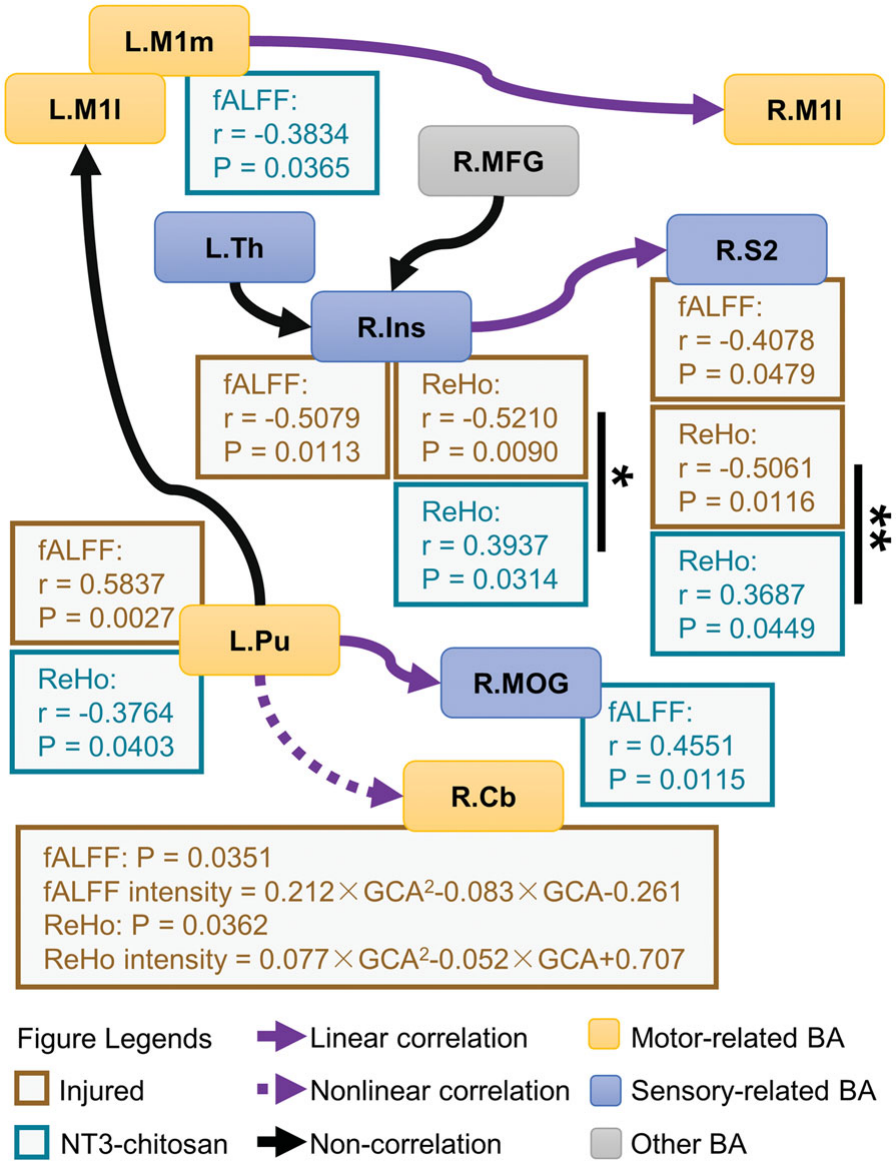
The injured and NT3-chitosan-treated animals have different relationships between the intensity of information flow and the property of the spontaneous activities. Correlations between the intensity of the information flow that was affected by time × group interaction and the fALFF/ReHo values in the brain regions corresponding to these flows were shown (*r* and *p* values are given). No pronounced relationship was observed between other information flows and the properties of local brain activity. Significant differences in correlation mode between the two groups were calculated (Chow-test with Bonferroni multiple comparisons). *p < 0.05; **p < 0.01. L: left; R: right; M1l: lateral primary motor cortex; M1m: medial primary motor cortex; Th: thalamus; MFG: middle frontal gyrus; Ins: insula; S2: secondary somatosensory cortex; Pu: putamen; MOG: middle occipital gyrus; Cb: cerebellum.

To further reveal the potential causes of alterations in brain functional interactive connections, we have evaluated the relationship between the intensity of information flow and the properties of the spontaneous activities (fALFF/ReHo) of local neuronal populations in the origin and recipient brain regions of information flow. Correlation analysis showed that the intensity of information flow from L.M1m, L.Pu, and right side insula (R.Ins) was related to the fALFF or ReHo signals of these regions. By contrast, the fALFF and ReHo of the right side secondary somatosensory cortex (R.S2) and right side middle occipital gyrus (R.MOG) in the sensory system components were related to the intensity of the received information flow (Figures 5 and S2). Since fALFF and ReHo reflected the intensity and homogeneity of spontaneous activities in local brain areas, respectively, these results proved that relationships existed between the partial causal interactions and the properties of the local activity in the origin and recipient regions. In the sensory information processing network, the ReHo at both ends of the information flow input from R.Ins to R.S2 was significantly correlated with information intensity. However, the correlation mode showed obvious variations between the two groups in the origin (F_1,51_=6.5523, *p*_corrected_ = 0.0268) and the recipient (F_1,51_=9.8836, *p*_corrected_ = 0.0056) areas, suggesting a different modulatory effect of R.Ins→R.S2 in the sensory network (Fig. 5). In the motor information processing network, the injured and NT3-chitosan groups showed independent “properties of local activity – intensity of information flow” relationships, indicating that spontaneous recovery and regenerative therapy exerted different effects on the reorganization of the motor network after SCI.

**Figure 6.**
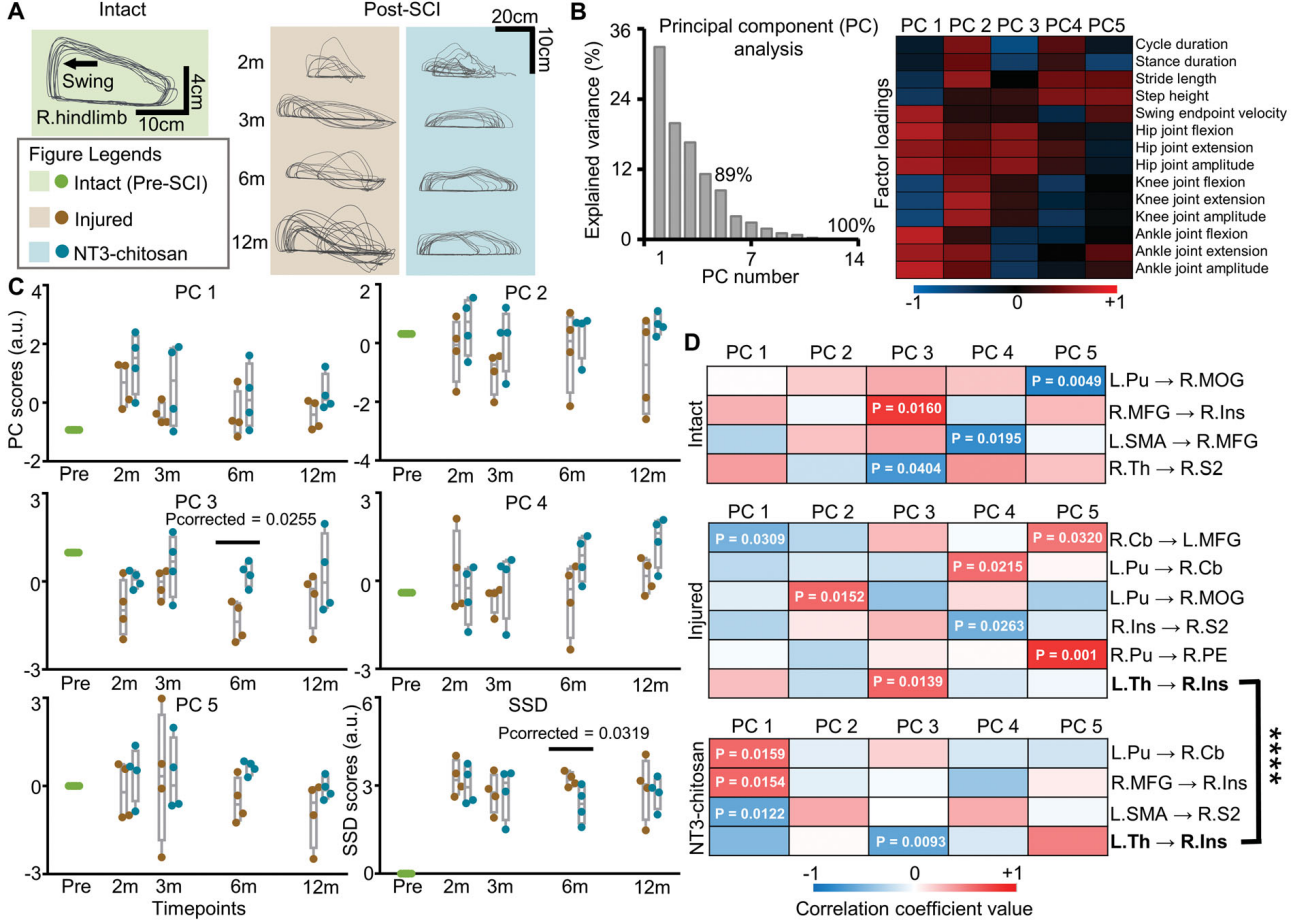
The injured and NT3-chitosan-treated animals have similar motor adjustments but different relationship modes between motor performance and information flow intensity. (A) Representative limb endpoint trajectories during consecutive stepping on a treadmill pre- and post-SCI showing the recovery of gait performance over time. (B) PCA was used to evaluate all parameters for each gait cycle. The first five PCs (PC1–5) with a cumulative variance interpretation rate of 89% were extracted. The factor loading matrix showed the correlation between each gait variable and each PC. Colour bar represents the value of correlation coefficients. (C) PC1-5 and SSD scores were compared between the two groups (independent t-test with Bonferroni multiple comparisons). SSD score indicated the extent of PC1-5 values which deviated from the intact. Significant differences were observed at 6 months post-SCI. (D) Significant relationships between PC1–5 scores and information flow intensity in animals with intact, injured, or NT3-chitosan treated status were displayed (*p* values have been given). The two groups showed diverse correlation patterns (Chow-test, ****F_1,29_ = 20.9057, *p* = 0.83 × 10^−4^). L: left; R: right; m: months; SSD: the sum of square deviation; Pu: putamen; MOG: middle occipital gyrus; MFG: middle frontal gyrus; Ins: insula; SMA: supplementary motor area; Th: thalamus; S2: secondary somatosensory cortex; Cb: cerebellum; PE: parieto-occipital association cortex.

We longitudinally evaluated the hindlimb stepping performance of injured and treated animals to assess the potential relationship between motor improvement and brain functional reorganization, and between motor improvement and fibre tracking results. The trajectory of the right (paralysed) hindlimb endpoint during consecutive walking showed the progressive recovery of gait capability over time (Figure 6(A)). Fourteen gait parameters were obtained from each gait cycle in each animal. PCA was then adopted to extract the most important information and reconstruct PCs1-5 in a single gait cycle (Figure 6(B)). PCA displayed that at 6 months post-SCI, the injured animals deviated from the intact gait pattern more than the NT3-chitosan treated animals (*p*_corrected_ = 0.0255) (Figure 6(C)). The SSD score was not 0 at all timepoints after operation, and no distinct variation was observed except 6 months post-SCI (*p*_corrected_=0.0319), which illustrated a similar degree of motor adjustment of the two groups in different pathophysiological states (Figure 6(C)). Although the coupling strength from left side thalamus (L.Th) to R.Ins was significantly correlated with PC3 score in both the injured and treated states, the correlation mode was indeed different (*F*_1,29_=20.9057, *p* = 0.83 × 10^−4^), indicating the heterogeneity of brain causal network reorganizations under similar motor adjustments (Figure 6(D)). Marked correlations between the ratio of regenerated fibres to remote normal-like fibres and SSD scores were observed in both the injured (*p* = 0.0021) and treated (*p* < 0.0001) animals, suggesting a good correspondence between the morphology and functions (Figure S3).

## 4. Discussion

Given the weak capability of spontaneous regeneration after SCI in adult mammals, a large number of studies have focussed on multidisciplinary approaches to achieve spinal cord axonal regeneration. Although the reconstruction of the projection pathway between supraspinal centres and below the injury level is crucial, independent recovery from the regulation of the supraspinal centres remains difficult [44,45]. A critical problem that has been neglected in strategies for promoting spinal cord regeneration is the occurrence of large-scale brain functional reorganization and the regulation of motor function recovery. In this study, we used injured and NT3-chitosan treated nonhuman primates to detect changes in the brain causal interaction network and their relationship with motor performance after SCI. We found that the causal network reorganization of brain function that was induced by NT3-chitosan regenerative therapy differed from that induced by the spontaneous recovery of SCI. The difference was partly attributed to the heterogeneous “local spontaneous low-frequency fluctuation – information flow intensity” relationship modes between the two groups. In addition, we also reported a correlation between the brain causal network reorganizations and the overall movement performance changes, and revealed the distinct relationship modes between the injured and NT3-chitosan animals.

Some studies on large-scale brain functional reorganization after SCI have investigated the changes in resting-state functional connectivity in the sensorimotor network [10,13,46–48]. Other works on central nervous system injury [49,50] and peripheral nerve injury [12,51] have shown that intra- and/or inter-hemispheric resting-state functional connections may occur at all stages of spontaneous recovery or treatment. But no study has reported the dominant period of the two types of reorganizations. Sawada *et al.* [52] used GCA to reveal that information flow between the nucleus accumbens and M1 cortex in the hemispheres of rhesus monkeys with cervical SCI increases in the early stage and decreases in the late stage. However, their study was limited to specific causal connections. Another cortical EEG study on nonhuman primates with partial SCI revealed that grasping-related signals exhibit increased interaction within the hemisphere and then gradually return to normal, whereas signals related to motor-preparation manifest inter-hemispheric interactions that gradually increase in intensity with time [53]. These results showed that the intensity of an interaction related to a specific action is high in the intra-hemisphere at the early stage (about 50 days) and high in the inter-hemisphere at the later stage (up to 150 d). In this study, we longitudinally evaluated the changes in resting-state information flow among multiple sensorimotor-related brain regions and revealed a model of “early intra-hemispheric reorganization dominant, late inter-hemispheric reorganization dominant” in the injured animals. Our results further expanded the conclusions obtained from previous studies on specific information flow and specific actions and provided new evidence for an in-depth description of the properties of brain plasticity. The combined results of previous reports and the opposing change patterns of injured and treated animals observed in this study suggested that the intra-/inter-hemispheric reorganization of brain causal interactions had a dominant stage that was unrelated to specific actions; its timing, however, was affected by therapeutic interventions. Our previous studies reported that NT3-chitosan materials could reduce inflammatory responses [28] and steadily release NT3 *in vivo* for 3 months [27]. The attenuated inflammatory response is beneficial for alleviating axonal degeneration and neuronal loss in the brain [54], which, together with the high concentrations of neurotrophic factors, may induce greater synaptic efficacy and axonal sprouting [55,56] to promote inter-hemispherical reorganizations at the early-stage after SCI.

Given that the lesion of nervous tissue could cause changes in brain function, we hypothesized that the regeneration should also lead to the reorganization of brain function with different characteristics. Our findings showed that this assumption was not entirely correct. Although the results showed almost independent processes of brain function reorganization under two kinds of spinal cord pathophysiology, the overall change degree of brain causal interaction networks was similar after 2 months post-SCI. This lack of distinction suggested that in the later stage of intervention therapy, even if the defective spinal cord neural tissue had regenerated, this process remained unable to reverse the alteration in the brain functional network. The reason for this phenomenon is twofold. First, the regeneration of nervous tissue does not indicate that the original contact pathways between the supraspinal centres and below the injury level are completely accurately reproduced; second, the newly constructed multi-synaptic pathway in the progress of regenerative therapy [26,29], as well as the simultaneous spontaneous recovery, may introduce abnormal projections [57]. It should be noted that our previous reports demonstrated that NT3-chitosan could induce not only axonal regeneration [25] but also robust neurogenesis from endogenous neural stem cells [29] in SCI rats. In this study, it is possible to speculate that NT3-chitosan treatment would have the same effect. Thus, the alteration of brain reorganization in the treated monkeys may be attributed to the results of regenerated axons together with new neurons. But further biological experiments are still needed.

Previous studies on peripheral nerve injury and regeneration have shown that injured animals can restore the intact brain function maps of limb movement after achieving highly specific innervation to the appropriate target organs [58]. By contrast, incorrect fibre–target organ connections present persistent abnormal activation of the brain region [59]. These studies described the effects on brain plasticity after the recovery of blocked information based on the active characteristics of local brain regions. The results of this study further indicated that not only the activation of local brain regions was abnormal but that large-scale causal interaction network among sensorimotor-related multiple brain regions also showed lasting adjustments. Since precise reconstruction of the disrupted projection pathway is currently difficult to achieve, the design of functional recovery strategies should take into account the effects induced by the lasting adjustments of brain networks.

NT3-chitosan treat animals showed a higher proportion of causal interactions originating from the left cortical areas than that in injured ones. The left-dominant in the treated group may due to the circuit repair in the right spinal cord by NT3-Chitosan transplantation. Another noteworthy result is that injured-only and treatment after the SCI acted on the information flow from R.Ins to R.S2. The insula is related to the processing and integration of somatosensory information [60]. The causal relationship between R.Ins and R.S2 was affected by the interruption of sensory afferents in the right side of the spinal cord, also demonstrating a good unilateral correspondence between the two. However, the varied coupling relationship of the intensity of information flow and the activity properties of the local brain area suggested that NT3-chitosan regenerative therapy and spontaneous recovery had different nociceptive afferent recovery patterns. In addition, a recent activity-dependent cell labelled mice study proved that insular cortex neurons can be activated during peripheral inflammation [61]. It is known that SCI would induce inflammatory response [62] but NT3-chitosan transplantation could reduce it [28]. The distinct coupling relationship between the injured and treated animals in the R.Ins may be regulated by different degrees of inflammation. However, the influence of inflammation-activated neurons in the insula on causal interactions still requires intensive study to identify.

It was previously shown that primates (monkeys and humans) can achieve substantially better spontaneous motor recovery than rodents after unilateral SCI by alternative circuits [63]. Many previous studies have also demonstrated the functional improvements induced by the post-injury plasticity after incomplete SCI [64–66]. Structural reorganizations, such as synaptic remodelling, dendritic spine growth and axonal sprouting and circuit reconstruction in the supra- and sub-lesional cord can restore functions after injury [67–69]. Since no therapy intervention has been performed in injured monkeys, an obvious recovery of stepping ability in this study can partially be attributed to the neural compensation mediated by above structural reorganizations. The recovery weakened the differences in gait performances between the injured and NT3-chitosan-treated monkeys, which may account for the ultimately similar motor adjustments between the two groups in this study. The coupling strength from L.Th to R.Ins was correlated with gait performance in both the spontaneous recovery and regenerative therapy animals, suggesting that it has potential as a regulation target for gait performance in a wide spectrum of spinal cord pathophysiology. However, the heterogeneous relationship indicates that the two groups have different GCA substrates for similar motor adjustments, suggesting distinct modes of regulations. Therefore, it may be difficult to achieve a desired effect in therapy by simply applying the regulation targets extracted from the purely injury. Our findings may be valuable for the development of rehabilitation protocols that modulate information flow to improve motor performance under various SCI interventions. Comparing the different effects of spontaneous recovery and treatment intervention on brain functional reorganization and their relationship with motor performance may enhance the extraction of the unique effects of treatment interventions and provide important guidance for improving therapeutic approaches.

Some limitations remain. First, a hemicord excision animal model was used in consideration of animal ethics. Residual spinal cord nerve tissues might have weakened the degree of impact on the brain functional network of injured animals and the difference in reorganizations and gait performances between the injured and regenerative therapy animals. Further studies can use complete SCI animal models to eliminate the possible effects of pathway compensation and to maximize the difference between injury and regeneration. Second, when the same brain region receives multiple information flows from different brain regions, the local activity of the receiving brain region may experience multiple effects, thus showing mixed results. This phenomenon complicates revealing the relationship between the intensity of a particular information flow and the properties of local activity at the recipient region. In addition, the further combination of histopathology and electrophysiological techniques is conducive to the in-depth analysis of the potential regulatory mechanism of regenerated tissue on brain functional reorganizations.

## 5. Conclusions

To the best of our knowledge, this is the first *in vivo* study that demonstrated distinct differences in brain plasticity between regenerative intervention and spontaneous recovery after SCI. The variations between these processes were mainly reflected in different spatiotemporal features, change trends of information flow intensities, and coupling relationships between the local neuronal activity properties and casual intensity. Furthermore, our results revealed correlations between walking recovery and partial GCA measures, proving a heterogeneous “information flow – motor performance” relationship between injured and treated animals at similar motor adjustments. Our findings suggested that regenerative therapy after SCI could lead to a unique pattern of brain functional reorganization. An accurate description of the interactive connections in the sensorimotor cortex and its relationship with motor performance will allow us to further understand the way regenerative therapy regulates brain functional networks and to evaluate the effect of regeneration with increased precision, which may contribute to the improvement in the intervention design and follow-up of spinal cord repair.

## Supplementary Material

Supplemental Material

## Data Availability

The data that support the findings of this study are available upon reasonable request.

## References

[1] Darian-Smith C. Synaptic plasticity, neurogenesis, and functional recovery after spinal cord injury. Neuroscientist. 2009;15(2):149–165. 19307422 10.1177/1073858408331372 PMC2897707

[2] Freund P, Weiskopf N, Ashburner J, et al. MRI investigation of the sensorimotor cortex and the corticospinal tract after acute spinal cord injury: a prospective longitudinal study. Lancet Neurol. 2013;12(9):873–881. 23827394 10.1016/S1474-4422(13)70146-7 PMC3744750

[3] Ghosh A, Haiss F, Sydekum E, et al. Rewiring of hindlimb corticospinal neurons after spinal cord injury. Nat Neurosci. 2010;13(1):97–104. 20010824 10.1038/nn.2448

[4] Rao JS, Ma M, Zhao C, et al. Atrophy and primary somatosensory cortical reorganization after unilateral thoracic spinal cord injury: a longitudinal functional magnetic resonance imaging study. Biomed Res Int. 2013;2013:753061. 24490171 10.1155/2013/753061 PMC3891744

[5] Sydekum E, Ghosh A, Gullo M, et al. Rapid functional reorganization of the forelimb cortical representation after thoracic spinal cord injury in adult rats. NeuroImage. 2014;87:72–79. 24185021 10.1016/j.neuroimage.2013.10.045

[6] Wrigley PJ, Press SR, Gustin SM, et al. Neuropathic pain and primary somatosensory cortex reorganization following spinal cord injury. Pain. 2009;141(1-2):52–59. 19027233 10.1016/j.pain.2008.10.007

[7] Rao JS, Ma M, Zhao C, et al. Fractional amplitude of low-frequency fluctuation changes in monkeys with spinal cord injury: a resting-state fMRI study. Magn Reson Imaging. 2014;32(5):482–486. 24629510 10.1016/j.mri.2014.02.001

[8] Rao JS, Ma M, Zhao C, et al. Alteration of brain regional homogeneity of monkeys with spinal cord injury: a longitudinal resting-state functional magnetic resonance imaging study. Magn Reson Imaging. 2015;33(9):1156–1162. 26117702 10.1016/j.mri.2015.06.011

[9] Chen Q, Zheng W, Chen X, et al. Whether visual-related structural and functional changes occur in brain of patients with acute incomplete cervical cord injury: a multimodal based MRI study. Neurosci. 2018;393:284–294. 10.1016/j.neuroscience.2018.10.014 30326291

[10] Hawasli AH, Rutlin J, Roland JL, et al. Spinal cord injury disrupts resting-state networks in the human brain. J Neurotrauma. 2018;35(6):864–873. 29179629 10.1089/neu.2017.5212 PMC5863102

[11] Nakanishi T, Nakagawa K, Kobayashi H, et al. Specific brain reorganization underlying superior upper limb motor function after spinal cord injury: a multimodal MRI study. Neurorehabil Neural Repair. 2021;35(3):220–232. 33514276 10.1177/1545968321989347

[12] Pawela CP, Biswal BB, Hudetz AG, et al. Interhemispheric neuroplasticity following limb deafferentation detected by resting-state functional connectivity magnetic resonance imaging (fcMRI) and functional magnetic resonance imaging (fMRI). NeuroImage. 2010;49(3):2467–2478. 19796693 10.1016/j.neuroimage.2009.09.054 PMC2818026

[13] Karunakaran KD, Yuan R, He J, et al. Resting-state functional connectivity of the thalamus in complete spinal cord injury. Neurorehabil Neural Repair. 2020;34(2):122–133. 31904298 10.1177/1545968319893299

[14] Rao JS, Liu Z, Zhao C, et al. Longitudinal evaluation of functional connectivity variation in the monkey sensorimotor network induced by spinal cord injury. Acta Physiol (Oxf). 2016;217(2):164–173. 26706280 10.1111/apha.12645

[15] Urbin MA, Royston DA, Weber DJ, et al. What is the functional relevance of reorganization in primary motor cortex after spinal cord injury? Neurobiol Dis. 2019;121:286–295. 30217521 10.1016/j.nbd.2018.09.009

[16] Goswami R, Anastakis DJ, Katz J, et al. A longitudinal study of pain, personality, and brain plasticity following peripheral nerve injury. Pain. 2016;157(3):729–739. 26588697 10.1097/j.pain.0000000000000430

[17] Rusanescu G, Mao J. Peripheral nerve injury induces adult brain neurogenesis and remodelling. J Cell Mol Med. 2017;21(2):299–314. 27665307 10.1111/jcmm.12965 PMC5264155

[18] Zhang CH, Ma ZZ, Huo BB, et al. Diffusional plasticity induced by electroacupuncture intervention in rat model of peripheral nerve injury. J Clin Neurosci. 2019;69:250–256. 31477463 10.1016/j.jocn.2019.08.088

[19] Davis KD, Taylor KS, Anastakis DJ. Nerve injury triggers changes in the brain. Neuroscientist. 2011;17(4):407–422. 21531986 10.1177/1073858410389185

[20] Rosenzweig ES, Brock JH, Lu P, et al. Restorative effects of human neural stem cell grafts on the primate spinal cord. Nat Med. 2018;24(4):484–490. 29480894 10.1038/nm.4502 PMC5922761

[21] Shaw DK, Saraswathy VM, Zhou LL, et al. Localized EMT reprograms glial progenitors to promote spinal cord repair. Dev Cell. 2021;56(5):613–626. 33609461 10.1016/j.devcel.2021.01.017 PMC8044706

[22] Babaloo H, Ebrahimi-Barough S, Derakhshan MA, et al. PCL/gelatin nanofibrous scaffolds with human endometrial stem cells/Schwann cells facilitate axon regeneration in spinal cord injury. J Cell Physiol. 2019;234(7):11060–11069. 30584656 10.1002/jcp.27936

[23] Nordblom J, Persson JKE, Svensson M, et al. Peripheral nerve grafts in a spinal cord prosthesis result in regeneration and motor evoked potentials following spinal cord resection. Restor Neurol Neurosci. 2009;27(4):285–295. 19738322 10.3233/RNN-2009-0478

[24] Alvarez Z, Kolberg-Edelbrock AN, Sasselli IR, et al. Bioactive scaffolds with enhanced supramolecular motion promote recovery from spinal cord injury. Science. 2021;374(6569):848–856. 34762454 10.1126/science.abh3602 PMC8723833

[25] Li XG, Yang ZY, Zhang AF, et al. Repair of thoracic spinal cord injury by chitosan tube implantation in adult rats. Biomaterials. 2009;30(6):1121–1132. 19042014 10.1016/j.biomaterials.2008.10.063

[26] Rao JS, Zhao C, Zhang AF, et al. NT3-chitosan enables de novo regeneration and functional recovery in monkeys after spinal cord injury. Proc Natl Acad Sci USA. 2018;115(24):E5595–5604. 29844162 10.1073/pnas.1804735115 PMC6004491

[27] Yang ZY, Duan HM, Mo LH, et al. The effect of the dosage of NT-3/chitosan carriers on the proliferation and differentiation of neural stem cells. Biomaterials. 2010;31(18):4846–4854. 20346501 10.1016/j.biomaterials.2010.02.015

[28] Duan HM, Ge WH, Zhang AF, et al. Transcriptome analyses reveal molecular mechanisms underlying functional recovery after spinal cord injury. Proc Natl Acad Sci USA. 2015;112(43):13360–13365. 26460053 10.1073/pnas.1510176112 PMC4629389

[29] Yang ZY, Zhang AF, Duan HM, et al. NT3-chitosan elicits robust endogenous neurogenesis to enable functional recovery after spinal cord injury. Proc Natl Acad Sci USA. 2015;112(43):13354–13359. 26460015 10.1073/pnas.1510194112 PMC4629318

[30] Babu RS, Muthusamy R, Namasivayam A. Behavioural assessment of functional recovery after spinal cord hemisection in the bonnet monkey (Macaca radiata). J Neurol Sci. 2000;178(2):136–152. 11018706 10.1016/s0022-510x(00)00394-4

[31] Rao JS, Liu Z, Zhao C, et al. Ketamine changes the local resting-state functional properties of anesthetized-monkey brain. Magn Reson Imag. 2017;43:144–150. 10.1016/j.mri.2017.07.025 28755862

[32] Rao JS, Liu Z, Zhao C, et al. Image correction for diffusion tensor imaging of rhesus monkey thoracic spinal cord. J Med Primatol. 2019;48(6):320–328. 31148186 10.1111/jmp.12422

[33] Rohlfing T, Kroenke CD, Sullivan EV, et al. The INIA19 template and NeuroMaps atlas for primate brain image parcellation and spatial normalization. Front Neuroinform. 2012;6:27. 23230398 10.3389/fninf.2012.00027 PMC3515865

[34] Mantini D, Gerits A, Nelissen K, et al. Default mode of brain function in monkeys. J Neurosci. 2011;31(36):12954–12962. 21900574 10.1523/JNEUROSCI.2318-11.2011 PMC3686636

[35] Hatanaka N, Nambu A, Yamashita A, et al. Somatotopic arrangement and corticocortical inputs of the hindlimb region of the primary motor cortex in the macaque monkey. Neurosci Res. 2001;40(1):9–22. 11311401 10.1016/s0168-0102(01)00210-3

[36] Rao JS, Zhao C, Yang ZY, et al. Diffusion tensor tractography of residual fibers in traumatic spinal cord injury: a pilot study. J Neuroradiol. 2013;40(3):181–186. 23428240 10.1016/j.neurad.2012.08.008

[37] Zhao C, Rao JS, Pei XJ, et al. Diffusion tensor imaging of spinal cord parenchyma lesion in rat with chronic spinal cord injury. Magn Reson Imaging. 2018;47:25–32. 29154896 10.1016/j.mri.2017.11.009

[38] Zhao C, Rao JS, Pei XJ, et al. Longitudinal study on diffusion tensor imaging and diffusion tensor tractography following spinal cord contusion injury in rats. Neuroradiology. 2016;58(6):607–614. 26931783 10.1007/s00234-016-1660-7

[39] Van den Brand R, Heutschi J, Barraud Q, et al. Restoring voluntary control of locomotion after paralyzing spinal cord injury. Science. 2012;336(6085):1182–1185. 22654062 10.1126/science.1217416

[40] Wei RH, Zhao C, Rao JS, et al. Neuromuscular control pattern in rhesus monkeys during bipedal walking. Exp Anim. 2019;68(3):341–349. 30930341 10.1538/expanim.18-0180 PMC6699981

[41] Wei RH, Zhao C, Rao JS, et al. The kinematic recovery process of rhesus monkeys after spinal cord injury. Exp Anim. 2018;67(4):431–440. 29769463 10.1538/expanim.18-0023 PMC6219880

[42] Zhao C, Song W, Rao JS, et al. Combination of kinematic analyses and diffusion tensor tractrography to evaluate the residual motor functions in spinal cord-hemisected monkeys. J Med Primatol. 2017;46(5):239–247. 28543057 10.1111/jmp.12276

[43] Liang H, Gong X, Chen M, et al. Interactions between feedback and lateral connections in the primary visual cortex. Proc Natl Acad Sci USA. 2017;114(32):8637–8642. 28739915 10.1073/pnas.1706183114 PMC5559040

[44] Courtine G, Song B, Roy RR, et al. Recovery of supraspinal control of stepping via indirect propriospinal relay connections after spinal cord injury. Nat Med. 2008;14(1):69–74. 18157143 10.1038/nm1682 PMC2916740

[45] Han Q, Ordaz JD, Liu NK, et al. Descending motor circuitry required for NT-3 mediated locomotor recovery after spinal cord injury in mice. Nat Commun. 2019;10(1):5815. 31862889 10.1038/s41467-019-13854-3 PMC6925225

[46] Kaushal M, Oni-Orisan A, Chen G, et al. Evaluation of whole-brain resting-state functional connectivity in spinal cord injury: a large-scale network analysis using network-based statistic. J Neurotrauma. 2017;34(6):1278–1282. 27937140 10.1089/neu.2016.4649

[47] Min YS, Park JW, Jin SU, et al. Alteration of resting-state brain sensorimotor connectivity following spinal cord injury: a resting-state Functional Magnetic Resonance Imaging Study. J Neurotrauma. 2015;32(18):1422–1427. 25945389 10.1089/neu.2014.3661

[48] Oni-Orisan A, Kaushal M, Li W, et al. Alterations in cortical sensorimotor connectivity following complete cervical spinal cord injury: a prospective resting-state fMRI study. PLoS One. 2016;11(3):e0150351. 26954693 10.1371/journal.pone.0150351 PMC4783046

[49] Hou J, Xiang Z, Yan R, et al. Motor recovery at 6 months after admission is related to structural and functional reorganization of the spine and brain in patients with spinal cord injury. Hum Brain Mapp. 2016;37(6):2195–2209. 26936834 10.1002/hbm.23163 PMC6867385

[50] Matsubayashi K, Nagoshi N, Komaki Y, et al. Assessing cortical plasticity after spinal cord injury by using resting-state functional magnetic resonance imaging in awake adult mice. Sci Rep. 2018;8(1):14406. 30258091 10.1038/s41598-018-32766-8 PMC6158265

[51] Pelled G, Chuang KH, Dodd SJ, et al. Functional MRI detection of bilateral cortical reorganization in the rodent brain following peripheral nerve deafferentation. NeuroImage. 2007;37(1):262–273. 17544301 10.1016/j.neuroimage.2007.03.069 PMC2253720

[52] Sawada M, Kato K, Kunieda T, et al. Function of the nucleus accumbens in motor control during recovery after spinal cord injury. Science. 2015;350(6256):98–101. 26430122 10.1126/science.aab3825

[53] Chao ZC, Sawada M, Isa T, et al. Dynamic reorganization of motor networks during recovery from partial spinal cord injury in monkeys. Cereb Cortex. 2019;29(7):3059–3073. 30060105 10.1093/cercor/bhy172

[54] Zhao C, Bao SS, Xu M, et al. Importance of brain alterations in spinal cord injury. Sci Prog. 2021;104(3):368504211031117. 34242109 10.1177/00368504211031117 PMC10450736

[55] Darian-Smith C, Gilbert CD. Axonal sprouting accompanies functional reorganization in adult cat striate cortex. Nature. 1994;368(6473):737–740. 8152484 10.1038/368737a0

[56] Florence SL, Taub HB, Kaas JH. Large-scale sprouting of cortical connections after peripheral injury in adult macaque monkeys. Science. 1998;282(5391):1117–1121. 9804549 10.1126/science.282.5391.1117

[57] Navarro X, Vivó M, Valero-Cabré A. Neural plasticity after peripheral nerve injury and regeneration. Prog Neurobiol. 2007;82(4):163–201. 17643733 10.1016/j.pneurobio.2007.06.005

[58] Wall JT, Felleman D, Kaas JH. Recovery of normal topography in the somatosensory cortex of monkeys after nerve crush and regeneration. Science. 1983;221(4612):771–773. 6879175 10.1126/science.6879175

[59] Wall JT, Kaas JH. Long-term cortical consequences of reinnervation errors after nerve regeneration in monkeys. Brain Res. 1986;372(2):400–404. 3708369 10.1016/0006-8993(86)91153-4

[60] Taylor KS, Anastakis DJ, Davis KD. Cutting your nerve changes your brain. Brain. 2009;132(Pt 11):3122–3133. 19737843 10.1093/brain/awp231

[61] Koren T, Yifa R, Amer M, et al. Insular cortex neurons encode and retrieve specific immune responses. Cell. 2021;184(24):5902–5915. 34752731 10.1016/j.cell.2021.10.013

[62] Zrzavy T, Schwaiger C, Wimmer I, et al. Acute and non-resolving inflammation associated with oxidative injury after human spinal cord injury. Brain. 2021;144(1):144–161. 33578421 10.1093/brain/awaa360 PMC7880675

[63] Friedli L, Rosenzweig ES, Barraud Q, et al. Pronounced species divergence in corticospinal tract reorganization and functional recovery after lateralized spinal cord injury favors primates. Sci Transl Med. 2015;7(302):302ra134–ra302ra134. 10.1126/scitranslmed.aac5811 PMC5669362 26311729

[64] Filipp ME, Travis BJ, Henry SS, et al. Differences in neuroplasticity after spinal cord injury in varying animal models and humans. Neural Regen Res. 2019;14(1):7–19. 30531063 10.4103/1673-5374.243694 PMC6263009

[65] Marufa SA, Hsieh TH, Liou JC, et al. Neuromodulatory effects of repetitive transcranial magnetic stimulation on neural plasticity and motor functions in rats with an incomplete spinal cord injury: a preliminary study. PLoS One. 2021;16(6):e0252965. 34086836 10.1371/journal.pone.0252965 PMC8177618

[66] Rao JS, Zhao C, Bao SS, et al. MRI metrics at the epicenter of spinal cord injury are correlated with the stepping process in rhesus monkeys. Exp Anim. 2022;71:139–149. 34789621 10.1538/expanim.21-0154 PMC9130044

[67] Guerout N. Plasticity of the injured spinal cord. Cells. 2021;10:1886. 34440655 10.3390/cells10081886 PMC8395000

[68] Krupa P, Siddiqui AM, Grahn PJ, et al. The translesional spinal network and its reorganization after spinal cord injury. Neuroscientist. 2022;28(2):163–179. 33089762 10.1177/1073858420966276

[69] Sofroniew MV. Dissecting spinal cord regeneration. Nature. 2018;557(7705):343–350. 29769671 10.1038/s41586-018-0068-4

