# Supplementary material for: Neural regeneration therapy after spinal cord injury induces unique brain functional reorganizations in rhesus monkeys
Source: Ann Med. 2022 Jul 6;54(1):1867–83. doi: 10.1080/07853890.2022.2089728 (PMC9272921; doi:10.1080/07853890.2022.2089728)
Supplement: Supplemental Material [file IANN_A_2089728_SM5235.docx]

**Supplementary Information**


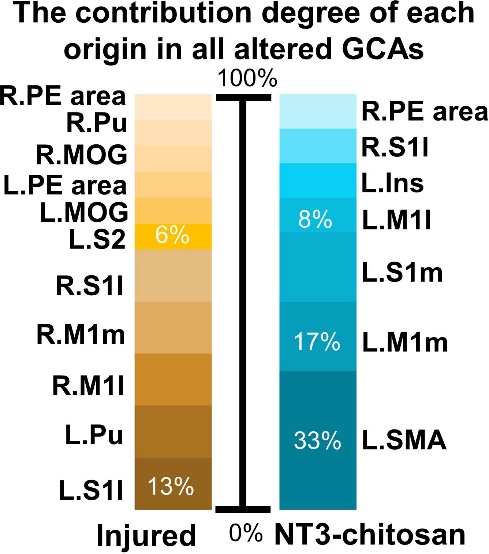


**Fig. S1** The proportion of GCA emitted from each origin with the number of GCA changed. L, left; R, right; S1l, lateral primary somatosensory cortex; M1l, lateral primary motor cortex; Pu, putamen; MOG, middle occipital gyrus; M1m, medial primary motor cortex; Ins, insula; PE area, parieto-occipital association cortex; S2, secondary somatosensory cortex; SMA, supplementary motor area; S1m, medial primary somatosensory cortex.


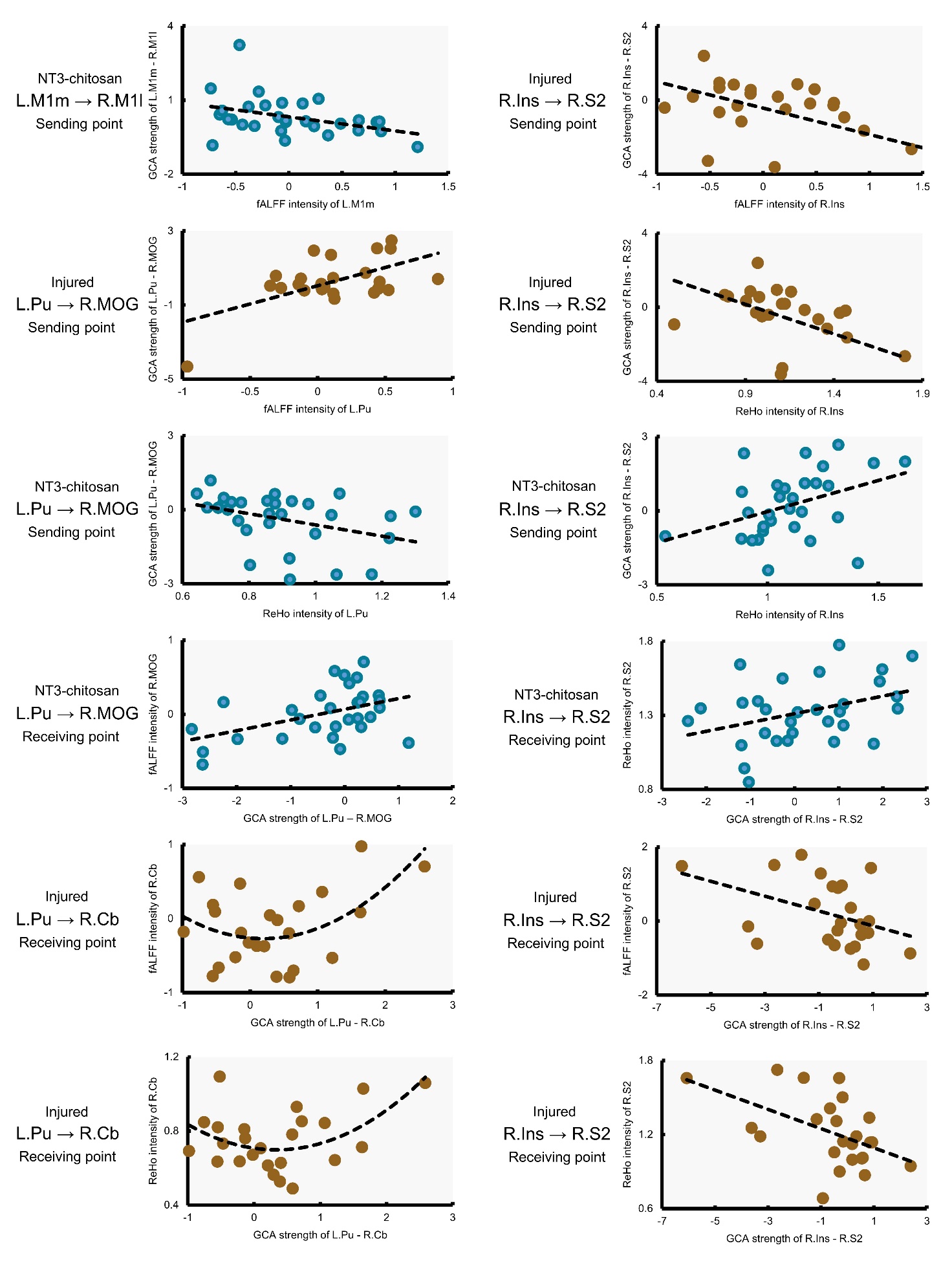


**Fig. S2** Relationship existed between the coupling strength and properties of the local activity in the origin and recipient brain regions. Data points (brown: injured animals; dark cyan: NT3-chitosan treated animals) represent the value of each monkey at each time point. Dashed lines are linear or quadratic fit lines. Correlation coefficients have been given in Fig. 5. L, left; R, right; M1l, lateral primary motor cortex; M1m, medial primary motor cortex; Th, thalamus; MFG, middle frontal gyrus; Ins, insula; S2, secondary somatosensory cortex; Pu, putamen; MOG, middle occipital gyrus; Cb, cerebellum.


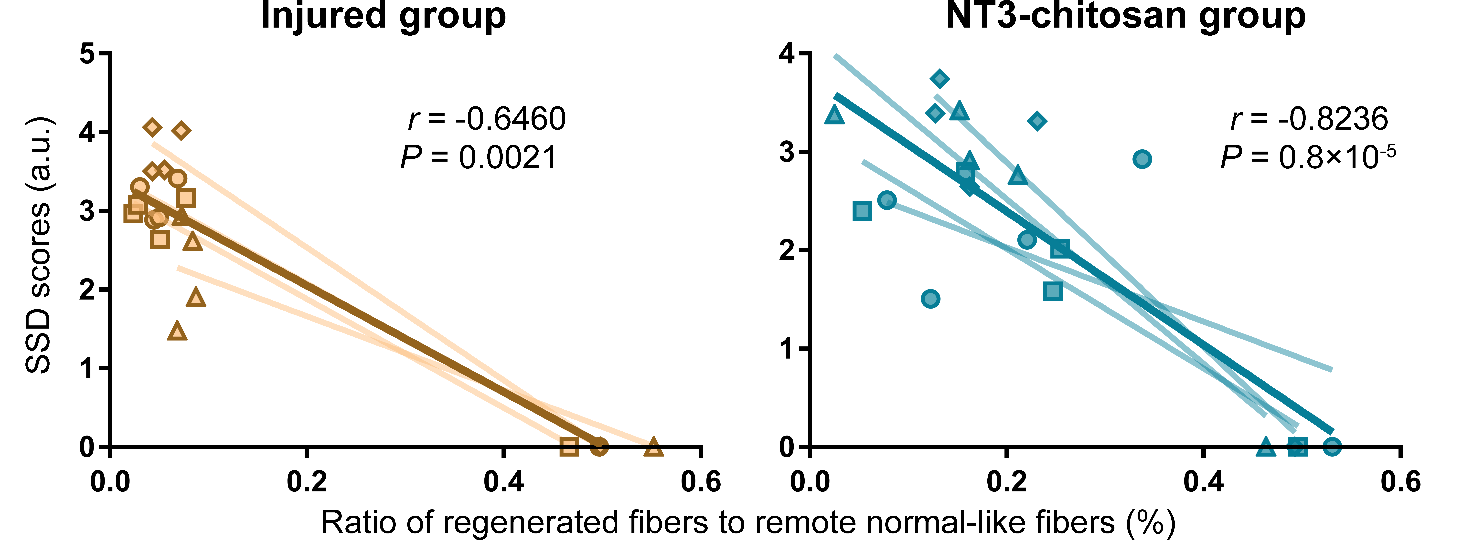


**Fig. S3** Relationship between the ratio of regenerated fibers to remote normal-like spinal cord fibers and SSD scores. Different symbols represent different individuals. Light/dark lines are linear fit lines of individual/group data, respectively. Correlation coefficient (r) and P values for group analysis are given. SSD, the sum of square deviation, indicated the extent of PC1-5 values which deviated from the intact.

**Supplementary Table 1. Time × Group repeated-measures ANOVA performed on information flows.**

| Information flow | F Statistic | P Value | Sample Main Effect of Group |
| --- | --- | --- | --- |
| L.SMA→R.MFG | F_4,24_ = 9.24 | P = 0.0491 | 12m: NT3-chitosan > Injured P = 0.0457 |
| R.MFG→R.Ins | F_1.55,9.28_ = 6.05 | P = 0.0257 | 1m: Injured > NT3-chitosan P = 0.0340 |
| L.SMA→R.S2 | F_4,24_ = 3.35 | P = 0.0259 | 2m: Injured > NT3-chitosan P = 0.0378 |
| L.SMA→R.Th | F_2.08,12.49_ = 10.46 | P = 0.0415 | 3m: Injured > NT3-chitosan P = 0.0125 |
| L.SMA→L.Th | F_2.08,12.50_ = 9.91 | P = 0.0447 | 2m: Injured > NT3-chitosan P = 0.0168 |
| L.Th→L.Ins | F_1.72,10.29_ = 17.21 | P = 0.0208 | 1m: Injured > NT3-chitosan P = 0.0034  6m: NT3-chitosan > Injured P = 0.0424 |
| L.Th→R.Ins | F_4,24_ = 2.86 | P = 0.0453 | 6m: NT3-chitosan > Injured P = 0.0312 |
| R.Th→R.S2 | F_4,24_ = 2.77 | P = 0.0503 | 3m: Injured > NT3-chitosan P = 0.0396 |
| R.Ins→R.S2 | F_1.76,10.58_ = 29.84 | P = 0.0095 | 3m: NT3-chitosan > Injured P = 0.0213 |
| R.Cb→L.MFG | F_4,24_ = 4.90 | P = 0.0049 | 1m: NT3-chitosan > Injured P = 0.0357  6m: NT3-chitosan > Injured P = 0.0154 |
| R.Cb→R.SMA | F_4,24_ = 5.28 | P = 0.0034 | 1m: NT3-chitosan > Injured P = 0.0348  3m: Injured > NT3-chitosan P = 0.0254 |
| L.Pu→L.M1l | F_4,24_ = 3.63 | P = 0.0189 | 1m: Injured > NT3-chitosan P = 0.0213 |
| L.Pu→R.Cb | F_1.58,9.49_ = 24.83 | P = 0.0123 | 1m: Injured > NT3-chitosan P = 0.0032 |
| L.Pu→R.MOG | F_4,24_ = 10.95 | P = 0.0390 | 1m: Injured > NT3-chitosan P = 0.0011 |
| R.MOG→L.MFG | F_4,24_ = 4.88 | P = 0.0050 | 1m: NT3-chitosan > Injured P = 0.0194 |
| R.Pu→R.PE area | F_1.82,10.89_ = 9.93 | P = 0.0445 | 2m: Injured > NT3-chitosan P = 0.0111 |
| L.M1m→R.M1l | F_1.52,9.14_ = 15.01 | P = 0.0252 | 2m: NT3-chitosan > Injured P = 0.0059  3m: NT3-chitosan > Injured P = 0.0326 |
| R.PE area→R.Pu | F_4,24_ = 5.04 | P = 0.0043 | 1m: Injured > NT3-chitosan P = 0.0193 |

All other interaction terms were non-significant. L, left; R, right; m, months; SMA, supplementary motor area; MFG, middle frontal gyrus; Ins, insula; S2, secondary somatosensory cortex; Th, thalamus; Cb, cerebellum; Pu, putamen; M1l, lateral primary motor cortex; MOG, middle occipital gyrus; PE area, parieto-occipital association cortex; M1m, medial primary motor cortex.
